# Emergency resection is an independent risk factor for decreased long-term overall survival in colorectal cancer: a matched-pair analysis

**DOI:** 10.1186/s12957-023-03182-8

**Published:** 2023-09-28

**Authors:** Katharina Esswein, Marijana Ninkovic, Elisabeth Gasser, Lars Barenberg, Alexander Perathoner, Reinhold Kafka-Ritsch

**Affiliations:** Department of Visceral, Transplant and Thoracic Surgery, Center of Operative Medicine, Medical University of Innsbruck, Innsbruck, Austria

**Keywords:** Colorectal cancer, Emergency, Resection, Recurrence, Overall survival

## Abstract

**Background:**

Colorectal cancer is one of the most common malignant neoplasms worldwide. Up to 30% of the patients present in an emergency setting despite an established screening program. Emergency colorectal resection is associated with increased mortality and morbidity as well as worse oncological outcome. This study aims to analyze the impact on tumor recurrence and survival in patients with an emergency colorectal resection, independent of sex, age, and tumor stage.

**Methods:**

Patients, who underwent an oncological resection for colorectal cancer at the Medical University of Innsbruck, Department of Visceral, Transplant and Thoracic Surgery, between January 2003 and December 2018 were analyzed retrospectively and screened for emergency resections. Matched pairs were formed to analyze the impact of emergency operations on long-term outcomes, considering tumor stage, sex, and age, comparing it with elective patients.

**Results:**

In total, 4.5% out of 1297 patients underwent surgery in an emergency setting. These patients had higher UICC (Union internationale contre le cancer) stages than elective patients. After matching the patients for age, sex, and tumor stage, emergency patients still had higher mortality. The incidence of recurrence was higher (47.5% vs. 25.4%, *p* = 0.003) and the 5-year overall survival decreased (35.6% vs. 64.4%, *p* < 0.001) compared to the matched patients with elective resection. Correcting for 90-day mortality still a reduction in the 5-year overall survival was demonstrated (44% vs. 70%, *p* = 0,001). The left-sided colon tumors were more common in the emergency group (45.8% vs. 25.4%, *p* = 0.006) and the rectal tumors in the elective one (21.2% vs. 3.4%, *p* = 0.002).

**Conclusion:**

Patients undergoing emergency resection for colorectal cancer have a decreased tumor-specific and overall survival compared to patients after elective resection, independent of age, sex, and tumor stage, even after correcting for 90-day mortality. These findings confirm the importance of colorectal cancer awareness and screening to reduce emergency resections.

## Background

Colorectal cancer (CRC) is the third most common malignant neoplasm worldwide and the second leading cause of cancer-related death [1]. The prognosis and survival rate depend mainly on the tumor stage. The 5-year survival rate in UICC (Union internationale contre le cancer) stage I is over 90% and decreases to approximately 60% in UICC stage III [2]. Despite an established screening program that improves early detection rates and therapy, up to 30% of CRC patients present as emergencies [3–5]. Patients who undergo emergency resection are more likely to be women [3, 6, 7].

Some studies describe that emergency patients are more likely to have more postoperative complications, higher short-term mortality, and decreased overall survival including worse oncological outcome [3, 5–7]. This could be related to a critically ill state of the patients presenting as emergencies with obstruction, perforation, or hemorrhage. The tumor stage is frequently advanced in these patients, leading to a worse oncological outcome with shorter disease-free survival [6, 8]. Furthermore, in the emergency setting, there could be a lack of colorectal specialist surgeon and the surgical treatment therefore might be associated with increased mortality and morbidity [7].

This study aims to analyze the impact on tumor recurrence and overall survival in patients, who underwent an emergency resection of colorectal cancer, independent of sex, age, and tumor stage.

## Methods

For this retrospective study, all patients undergoing resection for colorectal cancer at the Medical University of Innsbruck, Department of Visceral, Transplant and Thoracic Surgery, between January 2003 and December 2018 were evaluated. Data was collected from medical reports, operative reports, anesthesia protocols, and results of histological findings using the electronic health records (Klinisches Informationssystem, KIS).

Patients who presented as an emergency and underwent an immediate emergency surgery were classified as emergency. All the other patients were considered as elective. Variables included sex, age, ASA (American Society of Anesthesiologists) physical status classification system, and tumor anatomical site. The tumor stage was assessed by the TNM-classification based on the histopathological evaluation and computed tomography (CT) scan. The histological examination of the resected specimen comprised grading as well as vascular-, lymphangio-, or perineural-status. Only patients with a curative intended colorectal resection were included (macroscopic R0-resection). Resection was classified as performed by a surgeon with or without colorectal specialization. Surgeons were considered colorectal specialists if they were senior physicians in the colorectal department. These specialists have several years of colorectal surgical experience and are responsible for the colorectal training. Non-specialists were classified as senior physicians in visceral surgery with other subspecialties. The Clavien-Dindo classification was used to describe postoperative complications. In addition, the length-of-stay and adjuvant chemotherapy were documented. For outcome analysis, matched pairs were randomly assigned to the emergency patients in relation 2:1, selected out of the elective operated patients with the same sex, age group, and tumor stage. Outcome variables implied recurrence and overall survival (OS).

Statistical analyses were performed with the software SPSS (IBM SPSS Statistics 20; International Business Machines Corporation; Armonk, NY, USA). Group correlations for categorical variables were performed with the chi-squared test or the Fisher exact test. The Mann–Whitney *U* test was used for continuous variables. Survival was analyzed by the Kaplan–Meier Method. The *p* value of 0.05 or less was considered significant.

The local ethics committee approved the study (Votum 1128/2022).

## Results

### All patients

In total, 4.5% (*n* = 59) out of 1 297 patients, who underwent resection for CRC were classified as emergencies. The median (range) age of all patients was 69 (23–101) years. Female were 42.1% (*n* = 546) of the patients, who were significantly older than male patients, with a median (range) of 71 (31–101) years vs. 68 (23–97) years, respectively (*p* = 0.027). The age differed significantly between the emergency and all elective patients with a median (range) age of 76 years (32–94) and 69 years (23–101), respectively (*p* = 0.001). In the emergency group, 50.8% of the patients were female compared to 41.7% in the elective one (*p* = 0.164). The UICC stage was significantly higher in the emergency group (*p* = 0.013). Most commonly, the emergency patients presented with obstruction in 64.4% (*n* = 38), followed by perforation in 23.7% (free *n* = 7, iatrogenic *n* = 5, covered *n* = 2), suspicion of appendicitis in 5.1% (*n* = 3), invagination in 3.4% (*n* = 2), abscess in 1.7% (*n* = 1), and hemorrhage in 1.7% (*n* = 1). In 11.0% (13/118) of the elective cases, the diagnosis of CRC was made through a screening colonoscopy. In 71.2% (84/118), further investigation was performed due to symptoms such as hematochezia, changes in bowel habits, or abdominal pain. Table 1 shows baseline information on the emergency and elective patients.

**Table 1:** Baseline information of the emergency and elective patients

Baseline characteristics	Emergency patients *n* = 59 (%)	Elective patients *n* = 1 238 (%)	*p*-value
Sex			0.16
Female	30 (50.8)	516 (41.7)	
Male	29 (49.2)	722 (58.3)	
Age group in years			** < 0.05**
< 30	0	1 (1.7)	
30–39	1 (1.7)	28 (2.3)	
40–49	3 (5.1)	76 (6.1)	
50–59	4 (6.8)	176 (14.2)	
60–69	8 (13.6)	373 (30.1)	
70–79	23 (39.0)	313 (25.3)	
80–89	15 (25.4)	234 (18.9)	
> 90	5 (8.5)	37 (3.0)	
UICC			**0.01**
Stage I	3 (5.1)	304 (24.7)	
Stage II	16 (27.1)	349 (28.4)	
Stage III	30 (50.8)	291 (23.7)	
Stage IV	10 (16.9)	285 (23.2)	

### Matched pair analysis

For further analyses, 118 out of all 1238 elective patients were matched regarding sex, age group, and tumor stage and assigned to the 59 emergency patients (match 2:1).

There was no difference in the documented number of removed lymph nodes between the elective and emergency surgeries [16 (2–65); *n* = 112 vs. 16 (5–51); *n* = 55, respectively *p* = 0.386]. Positive resection margins (R1) were documented in two elective patients and one emergency patient. The histopathologic features tended to be worse in the emergency group with more lymphovascular invasion in 48.0% vs. 37.5% (*p* = 0.221) and perineural invasion in 31.6% vs. 12.1% (*p* = 0.086), without statistical significance. A venous invasion was found in 20.8% of emergency patients compared to 18.0% of elective patients (*p* = 0.684). The grading did not differ between the groups. Tables 2 and 3 show the anatomical tumor site regarding the emergency status and sex.

**Table 2:** Anatomical tumor sites for the emergency patients and the matched elective pair

Anatomical site	Emergency patients *n* = 59 (%)	Matched patients *n* = 118 (%)	*p*-value
Right-sided colon	30 (50.8)	63 (53.4)	0.749
Left-sided colon	27 (45.8)	30 (25.4)	**0.006**
Rectum	2 (3.4)	25 (21.2)	**0.002**

**Table 3:** Anatomical tumor site for female and male patients in the emergency group

Anatomical site	Female patients *n* = 30 (%)	Male patients *n* = 29 (%)	*p*-value
Right-sided colon	14 (46.7)	16 (55.2)	0.514
Left-sided colon	15 (50.0)	12 (41.4)	0.506
Rectum	1 (3.3)	1 (3.4)	0.981

A trend towards lower ASA scores (stage I and II) was seen in elective vs. emergency patients with 55.0% vs. 44.4%, respectively (*p* = 0.202). Men had significantly higher ASA scores (ASA III and IV) than women with 56.8% vs. 40.2%, respectively (*p* = 0.035). The median (range) length-of-stay was 16.5 days (5–124) in the elective and 18.0 days (1–54) in the emergency group (*p* = 0.990). No complication occurred in 57.6% of the elective and 52.5% of the emergency patients. Minor complications (Clavien-Dindo I and II) and major complications (Clavien-Dindo III-V) were documented for 20.3% and 22.0% of the elective, and 23.7% and 23.7% of the emergency patients. Death (Clavien-Dindo V) occurred in 3.4% of the elective and in 10.2% of the emergency group. The time between surgery and the start of adjuvant chemotherapy did not differ between the elective and emergency cases [median 1 (1–6) month *n* = 43 vs. 1 (1–7) month *n* = 24, respectively] or between female and male patients [median 1 (1–7) month *n* = 31 vs. 1 (1–6) month *n* = 36, respectively]. Elective resections were performed by surgeons with colorectal qualifications in 88.1% (104/118) and emergency resections in 78.0% (46/59), *p* = 0.076. The other resections were performed by a non-specialist. The resection was minimally invasive in 23.7% (28/118) of the elective cases and in 3.4% (2/59) of the emergency cases. Regarding the elective cases, the length-of-stay was significantly shorter in the minimally invasive group [median (range) 10.5 days (5–124) vs. 18 days (8–42), *p* = 0.001]. The time between surgery and the start of adjuvant chemotherapy did not differ between the groups [median (range) 1 month (1–6) for both groups]. In the emergency group, patients with an open approach had a median (range) length-of-stay of 18.5 days (9–33) and the time to adjuvant chemotherapy was 1 month (1–7). The two patients with minimally invasive approach stayed in the hospital for 12 and 22 days.

### Survival and recurrence

The mean follow-up for survival analysis was 4.7 years. Significantly more emergency patients had a recurrent or progressive disease compared to the matched elective patients (47.5% vs. 25.4%, *p* = 0.003) after a median (range) time of 12 months (1–70) and 15 months (2–78), respectively. The 5-year disease-free survival was 75.4% for elective and 54.2% for emergency patients (*p* < 0.001). Between women and men, there was no significant difference in recurrence (33.3% vs. 32.2%, *p* = 0.87) or in the 5-year disease-free survival (67.8% vs. 69.0%, *p* = 0.787). In the elective group, the recurrence tended to be higher after resection by a specialist vs. non-specialist (27.9% vs. 7.1%, *p* = 0.094). After emergency resection, the recurrence was slightly higher after resection by a non-specialist (53.8% vs. 45.7%,* p* = 0.601). The 5-year disease-free survival was in the elective group higher without significant difference after resection by a non-specialist (92.9% vs. 73.1%, *p* = 0.204) and in the emergency group higher after resection by a specialist (56.5% vs. 46.2%, *p* = 0.833), without significant difference.

**Fig. 1:**
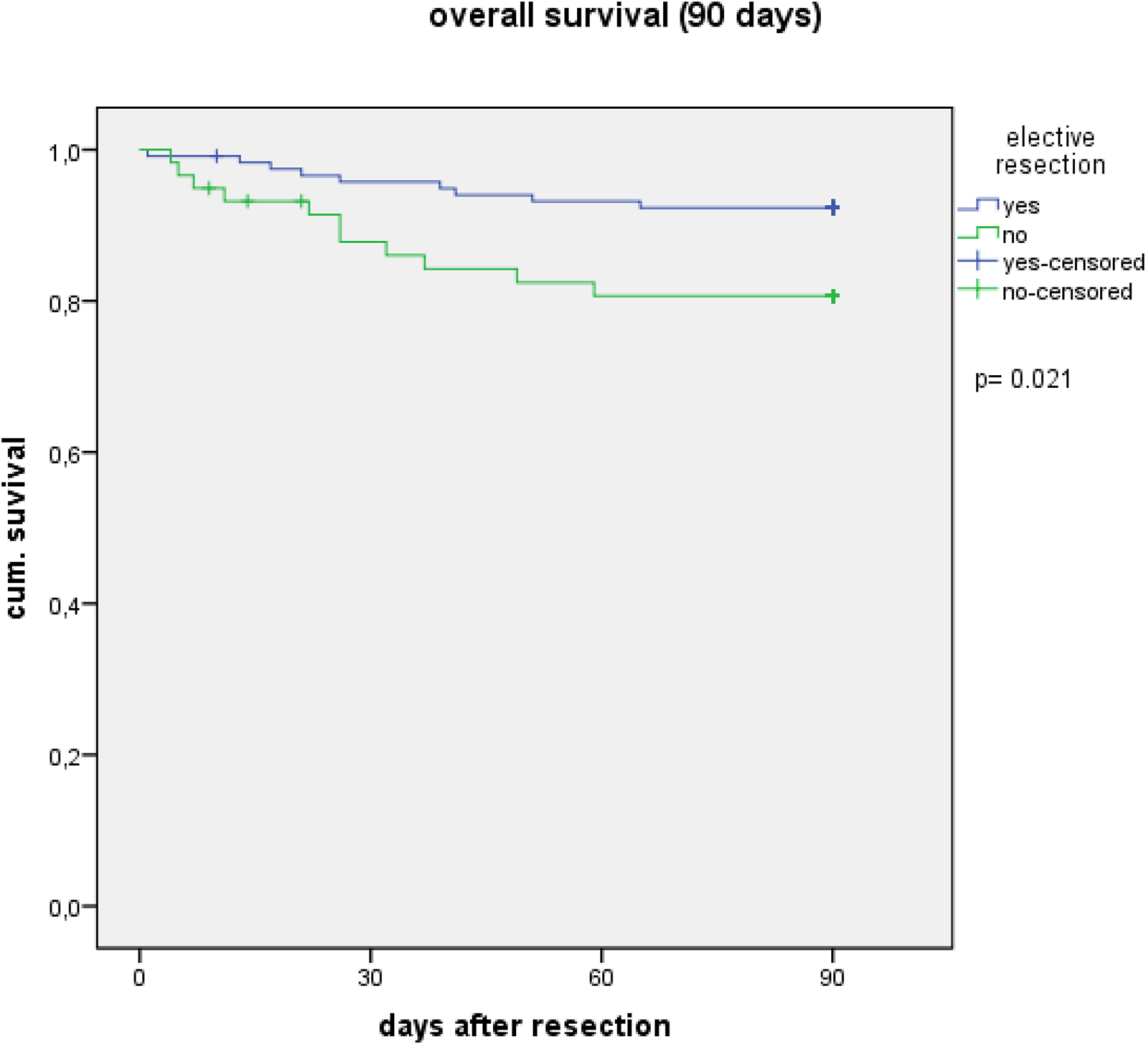
Overall survival in the first 90 days after resection

**Fig. 2:**
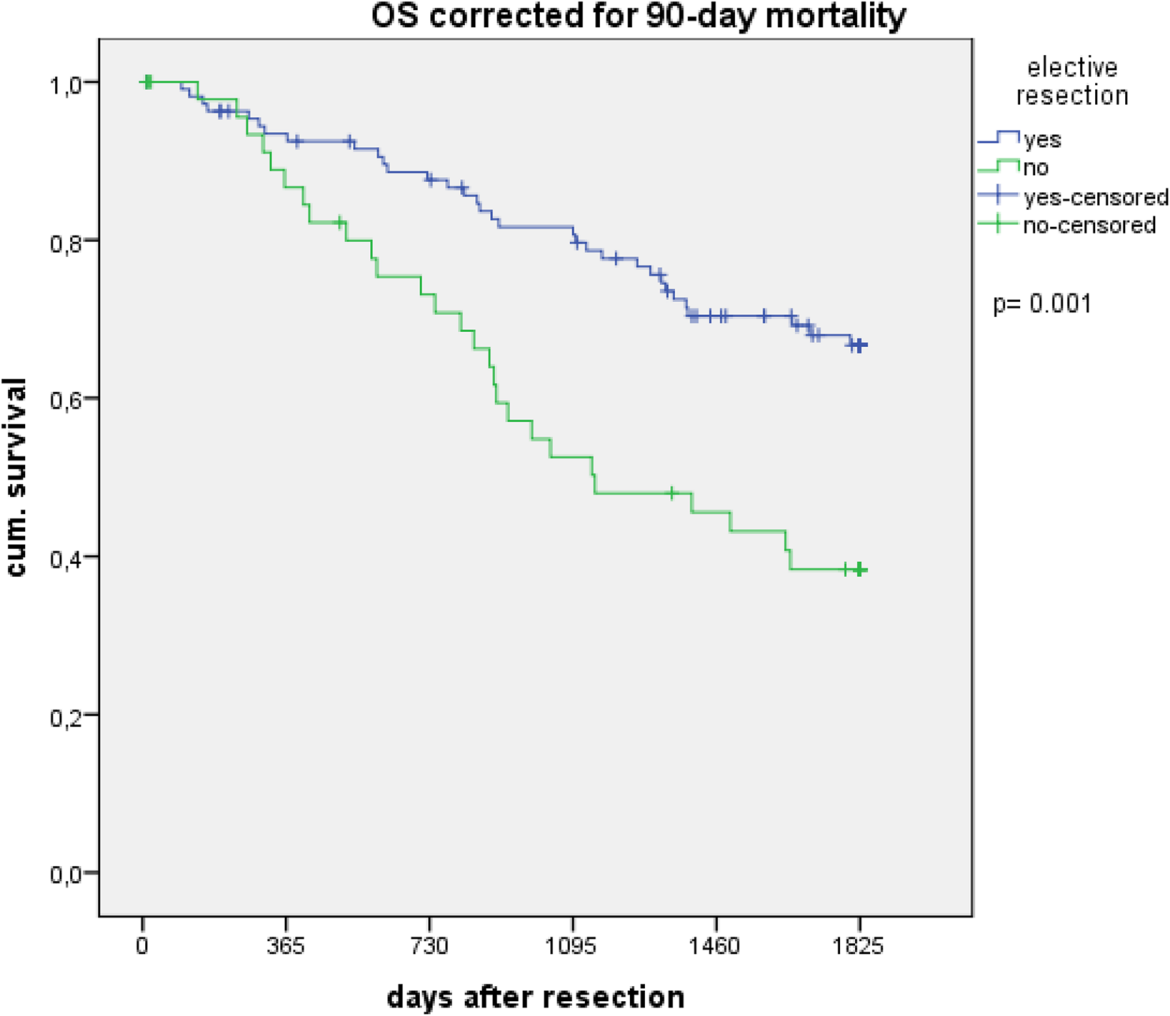
Overall survival corrected for 90-day mortality (patients, who died in the first 90 days postoperative were excluded for this analysis). OS, overall survival

The overall survival was lower in the emergency group compared to the elective with 35.6% vs. 64.4% (*p* < 0,001) after 5 years. Table 4 and Figs. 1 and 2 show the overall and long-term survival at different times. The overall survival of women tended to be worse in the elective group with a median of 77.7 vs. 105.7 months in men (*p* = 0.20) and better in the emergency group with 34.6 vs. 24.8 months in men (*p* = 0.58), without a significant difference. If the resection was performed by a colorectal specialist, the 5-year overall survival was higher with 39.1% vs. 23.1% (*p* = 0.623) in the emergency group and with 66.3% vs. 50.0% (*p* = 0.176) in the elective group, without significant difference. The 5-year overall survival was 71.4% after minimally invasive resection and 62.2% after open resection (*p* = 0.373).

**Table 4:** Overall survival of emergency and matched elective patients with additional correcting for 90-day mortality (patients, who died in the first 90 days postoperative were excluded)

	Overall survival		
	Emergency patients % (*n* = 59)	Matched patients % (*n* = 118)	*p*-value
30 days	88.1	95.8	0.051
90 days	81.4	92.4	0.021
1 year	72.9	86.4	0.017
3 years	45.8	75.4	< 0.001
5 years	35.6	64.4	< 0.001
	Overall survival corrected for 90-day mortality		
	Emergency patients % (*n* = 48)	Matched patients % (*n* = 109)	*p*-value
1 year	89.6	93.6	0.361
3 years	56.2	81.7	< 0.001
5 years	43.8	69.7	0.001

## Discussion

The study analyzed the outcome of patients, who underwent emergency resection for colorectal cancer, in relation to a matched pair cohort of patients with urgent or elective resection corrected for tumor stage, age, and sex.

Emergency presentations occurred in 4.5% in our cohort, which is lower than in most studies with a percentage of 10–15% [3, 4]. However, the number varies up to 30% in some studies due to different inclusion criteria [5]. We classified emergencies as patients undergoing immediate resection, which means only patients requiring emergency surgery, that could not be postponed. Regardless of the exact number, the emergency presentation and treatment of CRC patients are still challenging issues. They mostly present with obstruction, perforation, or hemorrhage [7, 8]. In our study, over 60% of the patients presented with obstruction, and the tumor was localized on the left side significantly more commonly in the emergency group. This is a typical pattern as the cecum and ascending colon have a larger diameter compared to the left-sided colon. Significantly fewer patients presented with rectal cancer as emergencies. An early symptom of rectal tumors is hematochezia or a disorder in defecation, resulting mostly in a medical examination before emergency presentation.

Frequently, emergency patients present in a critically ill state, leading to a higher risk of perioperative complications, longer length-of-stay, and worse outcome [9]. Our findings indicate that emergency patients tend to have higher ASA scores which are associated with increased postoperative complications and longer hospital stays, although the difference was not statistically significant. The complication rate and comparable prolonged hospital stay in the elective group may be attributed to the higher proportion of rectal resections in this group, known for their inherent complexity during the perioperative period and the additional time required for managing a protective stoma. Emergency patients had decreased survival, starting as early as 30 days after surgery and differing significantly after 90 days. Various studies describe decreased survival after emergency presentation. Xu et al. showed 69% greater odds of dying within 30 days of surgery for emergency patients, and in the study of Amri et al., the 30-day death rate was tenfold increased [5, 8]. In addition to the immediate postoperative period, emergency patients also experience worse long-term outcomes [3–5, 7, 8]. To evaluate the impact on long-term outcome, we conducted an analysis excluding patients, who dropped out in the first 90 days following resection. The results revealed a significant decrease in the 5-year overall survival for emergency patients, with a rate of 43.8% compared to 69.7% for elective patients. Hence, patients undergoing emergency resection have significantly poorer long-term survival, irrespectively of their short-term outcomes. The tumor stage has the biggest impact on survival, with significantly lower OS observed in higher stages compared to early stages, regardless of the advanced multimodal therapy today [2]. Patients with emergency presentation for CRC seem to have more advanced tumor stages and worse pathologic features [3, 8]. Considering the significant impact of tumor stage, we matched the patients based on tumor stage to describe the outcome independent of the tumor stage. Despite matching the data for tumor stage, we observed a decreased tumor-specific and overall survival for emergency patients, regardless of the tumor stage.

Age and sex are additional risk factors associated with worse outcomes [8, 10]. While the incidence of colorectal cancer is higher in men, female patients more frequently present as emergencies [3, 6, 7, 11]. One possible explanation is the higher rate of proximal colon cancer in women, which often appears as a flat lesion during colonoscopy, leading to a delayed diagnosis at a more advanced tumor stage [12]. In our cohort, female patients presented more commonly as emergencies, but a higher rate of right-sided colon could not be confirmed in the female group. Studies have failed to prove assumptions that women participate in colorectal cancer screening programs less frequently than men [13, 14]. However, participation in screening programs does decrease with age and the incidence of colorectal cancer in older populations, particularly among female patients, is notably high [10, 12]. Despite the higher frequency of emergency presentations and older age among women, several studies have reported better survival outcomes compared to men. Possible explanations for this difference include genetic factors (e.g., microsatellite instability), protective hormonal effects, and environmental factor [10, 11, 15, 16].

Older patients constitute a significant proportion of the emergency cases in our study. This can be attributed to their lower participation in screening programs and the higher incidence of CRC in advanced age. Besides that, the recent study of Bretthauer et al. showed that the risk reduction of CRC by screening colonoscopy is not as high as assumed in clinical guidelines [17].

Despite matching the data for the risk factors such as tumor stage, sex, and age, our study revealed a decreased overall survival following emergency presentation. Notable, a lack of specialized surgical teams at night or on weekends can have a significant impact on morbidity and mortality. Biondo et al. have described that an emergency operation performed by a colorectal specialist is associated with a significant impact on morbidity and mortality [18]. Some studies have reported a higher occurrence of inadequate removal of lymph nodes or positive resection margins in emergency patients [4, 5, 8]. Consistent with our findings, other studies also support the notion that there is no disparity between emergency and elective surgery, implying adequate oncological resection [3, 7]. This could be explained by the fact that most emergency resections are performed by a colorectal specialist in our hospital, even in the emergency setting. We observed a higher 5-year overall survival in patients with resection by a specialist, but this is maybe due to the small number of patients operated by non-specialists and did not reach statistical significance. In the elective group, the recurrence rate tended to be slightly less without significant difference after resection by a non-specialist. This could be explained by the fact that easier operations, like patients with early tumor stages or without any previous operation, are performed by surgeons without colorectal qualification, as this analysis is not corrected for tumor stage, tumor location, or age. However, various studies indicate that emergency patients are less likely operated on by a specialist, which must be considered when analyzing the outcome of emergency and elective resections [7, 8].

Analyzing postoperative treatment, we found that the time to start adjuvant chemotherapy was similar between the emergency and elective group, suggesting that the recovery after surgery was not prolonged and the cancer work-up was not delayed. A French study with 404 patients reported a longer time to start adjuvant chemotherapy; however, other studies showed no delay [3, 19, 20].

Nevertheless, emergency patients had a higher recurrence rate. Emergencies are often accompanied by increased inflammation markers at the time of presentation. Cancer-related systemic inflammation can influence tumor progression, including invasion and metastasis, and is associated with worse outcomes [21]. Preoperative scores including C-reactive protein and albumin have prognostic value for outcome [22]. Therefore, the increased perioperative inflammation in emergency patients could be another factor contributing to higher recurrence rates and worse outcomes.

Our analysis has some limitations. Due to the retrospective design, the results depend on the documented data quality. Additionally, the cohort size is small, necessitating caution in interpretation. The number of analyzed patients is small, because only patients with immediate need for surgery and with curative colorectal resection were selected as emergencies. The rate of minimal-invasive resection is comparably low, which could be due to the inclusion period starting in 2003. The strength of the study is that over a 15-year period, all consecutive patients were assessed, and the analysis was matched by age, sex, and tumor stage as well as corrected for 90-day mortality. Further studies with larger cohorts are required to investigate suggested risk factors for postoperative and oncological outcomes, including pathologic features, perioperative blood analyses and resections performed by non-specialists in colorectal surgery.

## Conclusion

Our data indicate that patients undergoing emergency resection for CRC experience decreased tumor-specific and overall survival compared to patients who undergo elective resection, independent of age, sex, and tumor stage, even after correcting for 90-day mortality. These findings underscore the importance of CRC awareness and screening to reduce the need of emergent resections.

## Data Availability

The datasets generated and/or analyzed during the current study are not publicly available due to privacy or ethical restrictions but are available from the corresponding author on reasonable request.

## Abbreviations

ASA: American Society of Anesthesiologists
CRC: Colorectal cancer
CT: Computed tomography scan
OS: Overall survival
UICC: Union internationale contre le cancer
